# A biologically plausible embodied model of action discovery

**DOI:** 10.3389/fnbot.2013.00004

**Published:** 2013-03-12

**Authors:** Rufino Bolado-Gomez, Kevin Gurney

**Affiliations:** Department of Psychology, Adaptive Behaviour Research Group, University of SheffieldSheffield, UK

**Keywords:** phasic dopamine, basal ganglia, reinforcement learning, synaptic plasticity, intrinsic motivation, action selection, operant behavior

## Abstract

During development, animals can spontaneously discover action-outcome pairings enabling subsequent achievement of their goals. We present a biologically plausible embodied model addressing key aspects of this process. The biomimetic model core comprises the basal ganglia and its loops through cortex and thalamus. We incorporate reinforcement learning (RL) with phasic dopamine supplying a sensory prediction error, signalling “surprising” outcomes. Phasic dopamine is used in a cortico-striatal learning rule which is consistent with recent data. We also hypothesized that objects associated with surprising outcomes acquire “novelty salience” contingent on the predicability of the outcome. To test this idea we used a simple model of prediction governing the dynamics of novelty salience and phasic dopamine. The task of the virtual robotic agent mimicked an *in vivo* counterpart (Gancarz et al., 2011) and involved interaction with a target object which caused a light flash, or a control object which did not. Learning took place according to two schedules. In one, the phasic outcome was delivered after interaction with the target in an unpredictable way which emulated the *in vivo* protocol. Without novelty salience, the model was unable to account for the experimental data. In the other schedule, the phasic outcome was reliably delivered and the agent showed a rapid increase in the number of interactions with the target which then decreased over subsequent sessions. We argue this is precisely the kind of change in behavior required to repeatedly present representations of context, action and outcome, to neural networks responsible for learning action-outcome contingency. The model also showed cortico-striatal plasticity consistent with learning a new action in basal ganglia. We conclude that action learning is underpinned by a complex interplay of plasticity and stimulus salience, and that our model contains many of the elements for biological action discovery to take place.

## 1. Introduction

How can animals acquire knowledge of their potential *agency* in the world—that is, a repertoire of actions enabling the achievement of their goals? Moreover, how can this be done spontaneously without the animal being instructed, or without having some overt, primary reward assigned to successful learning? In this case we talk of *action discovery*, and call the learning *intrinsically motivated* (Oudeyer and Kaplan, 2007). It is typical of the kind of action learning found in the young as they discover their ability to influence their environment (Ryan and Deci, 2000). We argue that an understanding of the biological solution to these problems will lay foundations for a robust and extensible solution to skill acquisition in artificial agents like robots. We now outline the theoretical, behavioral and neuroscientific background to the paper.

**Figure 1:**
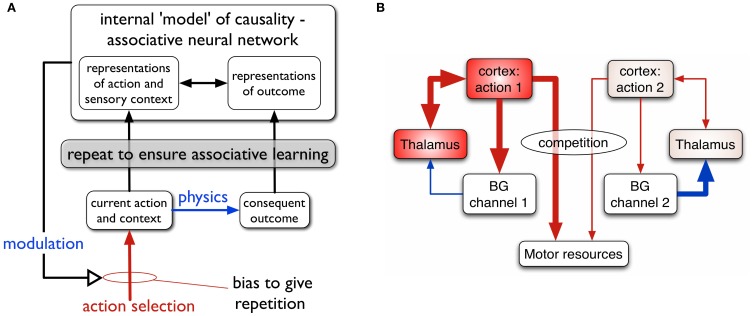
**(A)** Scheme for learning action-outcome associations—see text for details. **(B)** Loops through basal ganglia, thalamus, and cortex performing action selection in the animal brain. Two competing action channels are shown. The channel on the left encoding action 1 has a higher salience than that for channel 2. It has “won” the competition for behavioral expression in basal ganglia which has therefore released inhibition on its thalamic channel target, thereby allowing the corresponding thalamo-cortical loop to build up activity. Blue/red lines show inhibition/excitation, respectively and the width of lines encodes signal strength.

The relation between actions and outcomes is not a given—the animal must use reinforcement learning (RL) to acquire *internal models* of action-outcome contingencies associating context, action and outcome, and be able to deploy the relevant action given a context and a desired outcome or goal. Consider, for example, the act of switching on a particular room light. There is a *forward, prediction model*: “if I am in front of this switch and I press it, the light in the corner will come on.” There is also an *inverse model*: “if I need the light in the corner to come on, I need to press this switch here” (Gurney et al., 2013). The framework for action-outcome acquisition we propose is shown in Figure 1A.

We suppose that the internal models of action-outcome are encoded in associative neural networks. In order for these associations to be learned (possibly via some kind of Hebbian plasticity), representations of the motor action, sensory context, and the sensory outcome must be repeatedly activated in the relevant neural systems. This requires a transient change in the action selection propensities of the agent—its so-called selection *policy*—so that the to-be-learned action occurs more often than other competing actions. The repeated presentation of the representation of outcome is taken care of by physics; if the switch is pressed the agent doesn't have to do any more work to make the light come on.

The process of *repetition bias* in policy must continue until the new action-outcome has been learned, and then cease. We therefore require that the agent's policy is modulated by the predictions being developed in the forward model; as the outcome is predicted, the repetition bias must be reduced and, ultimately, removed. In general, we propose that the intrinsically motivated behavior is driven by novelty—the agent engages with the situation because the target object (e.g., the switch) is novel or that the “surprise” of the outcome on first encountering the light cause some plastic change in the policy engine.

In this paper, one of our aims is to understand the dynamics of repetition bias. To proceed, we therefore turn to the machinery for solving the problem of action selection, and policy encoding in the animal brain. We and others (Mink and Thach, 1993; Doya, 1999; Redgrave et al., 1999; Houk et al., 2007) have argued that a set of subcortical nuclei—the basal ganglia—are well placed to help solve this problem, and act as the policy engine or “actor” in the vertebrate brain.

The basal ganglia are connected in closed looped circuits with cortex, via thalamus (Figure 1B). Their outputs are tonically active and inhibitory, and selection is achieved by selectively releasing inhibition on cortico-thalamic targets that encode specific actions (Deniau and Chevalier, 1985). We refer to the neural representation of an action, and its anatomical instantiation, as it runs through these loops as an action *channel* (Redgrave et al., 1999). Release of inhibition on a thalamic channel allows activity in its corresponding thalamo-cortical loop to build up and eventually reach a threshold which allows behavioral expression of the action. More details of this architecture are given in the section 2.

Within this framework we can identify two components of a successfully established action encoding. First, within cortex, there must be the correct specific patterning of contextual (sensory, cognitive, and possibly homeostatic) and preparatory motor features. We refer to this as the *action request* and the overall level of activity in the action request is supposed to signal its urgency or *salience*. Channels within basal ganglia are subject to competitive processes therein and action requests with the highest salience are those that are selected. Clearly, one mechanism then for inducing repetition bias would be to enhance the salience of requests for the action to be discovered (Redgrave et al., 2011). A second component of action encoding occurs at the level of the main basal ganglia input nucleus—the striatum. Here, the cortical action request must selectively activate a subset of the striatal projection neurons, or so-called medium spiny neurons (MSNs). In this way, a striatal channel is established which can “listen” to the action request (Redgrave et al., 2011). For a neuron computing a weighted sum of inputs, this occurs by a process of matching the pattern of synaptic efficiencies to the strengths of action request components, resulting in a proportional encoding of salience. Evidence for such an encoding of salience in striatum has recently been provided by human fMRI studies (Zink et al., 2006).

To establish channel selectivity in MSNs requires cortico-striatal plasticity whose dynamics depend on the animal's behavior and resulting environmental feedback. The theory of RL encompasses exactly this scenario (Sutton and Barto, 1998) and so it is not surprising that cortico-striatal plasticity has been the subject of study using the classic algorithms of RL (such as temporal difference learning) with reinforcement contingent on biological reward. The reinforcement signal in this scenario is supposed to be supplied by short-latency phasic dopamine bursts which encode a *reward* prediction error (Schultz et al., 1997).

In contrast to this, we have recently argued that such signals are unlikely to be associated with primary reward as such, because they occur too soon to be the result of a relatively lengthy process of explicit evaluation in which the stimulus is assigned rewarding, neutral or aversive status. Instead, we propose that phasic dopamine primarily encodes a *sensory* prediction error which may be used to guide acquisition of goal-directed actions (Redgrave and Gurney, 2006; Redgrave et al., 2008).

This interpretation does not preclude a role for reward in modulating the phasic dopamine signal, and these issues are explored further in section 4.3 in the “Discussion.” However, under the sensory prediction error hypothesis, action acquisition is supposed to take place with the following sequence of events. An animal performs an action which results in an unexpected outcome. The phasic component of the outcome (not requiring computation of value) causes midbrain dopamine neurons to fire (Comoli et al., 2003) eliciting a phasic release of dopamine in striatum (the mechanistic substrate for this is described in more detail in section 2.5.4). This then acts to induce cortico-striatal plasticity associated with recently active action-based representations in cortex, and corresponding striatal responses. If repetition bias is operative, this sequence of events is repeated and MSNs in striatum can become selectively responsive to the action request which is required to elicit the environmental event. It is also possible that this plasticity can itself contribute to repetition bias, as each increment in the match between the patterns of synaptic strengths and action request should make the selection of the action more likely. However, one of the questions we address here is the extent to which this can be wholly responsible for transient policy changes seen *in vivo*. Fortunately there is a recent behavioral study (Gancarz et al., 2011) which provides data we can use to constrain the possibilities here.

At the neuronal level, electrophysiological data from studies in cortico-striatal plasticity have provided a complex and often confusing picture. Both long term depression (LTD) and long term potentiation (LTP) have been observed at glutamatergic (excitatory) cortical synapses on MSNs, and their expression is dependent on dopamine (Reynolds and Wickens, 2002; Calabresi et al., 2007). Further, this dependence is linked to specific dopamine receptor types in different populations of MSNs (Pawlak and Kerr, 2008) and has spike timing dependent characteristics (Fino et al., 2005; Pawlak and Kerr, 2008). This phenomenological complexity has hampered the development of a quantitative functional understanding of cortico-striatal plasticity. In particular, given the limitations of much *in vitro* data with regards to the class of MSNs based on their dopamine receptors, we would expect this data to display *mean* characteristics rather than those of one class alone. This is then necessarily reflected in models (Thivierge et al., 2007) which may account for spike timing and dopaminergic effects, but rely on data which is agnostic about the MSN classification.

Recently this impasse has been overcome in a study in striatal slices by Shen et al. (2008), in which the different classes of MSN could be reliably identified. In addition, this study deployed a variety of techniques to investigate the effects of dopamine depletion, thereby providing data at different levels of intrinsic dopamine. This study formed the basis of our recent spiking model of cortico-striatal plasticity (Gurney et al., 2009) which we adapt here for rate-coded neurons.

Within the framework described above, we seek to address in this study, the following questions about action discovery. Having proposed that action-outcome discovery depends on a repetition bias in selection policy, what are the mechanisms responsible for this? In particular what are the relative roles for enhanced cortical salience (“louder action request”), and better cortico-striatal transmission (“listening to the request”) induced by dopamine modulated cortico-striatal plasticity? If increased cortical salience is required, what is its origin? How should salience and plasticity be moderated by the development of the prediction model? Is any cortico-striatal plasticity observed in the model consistent with the requirements of long term afferent/synaptic-strength pattern matching? To ensure a biologically plausible solution, we take advantage of recent behavioral data (Gancarz et al., 2011), made possible with our embodied (robotic) approach, and recent *in vitro* data (Shen et al., 2008) on cortico-striatal plasticity.

## 2. Materials and methods

### 2.1. *In vivo* experimental comparison

**Figure 2:**
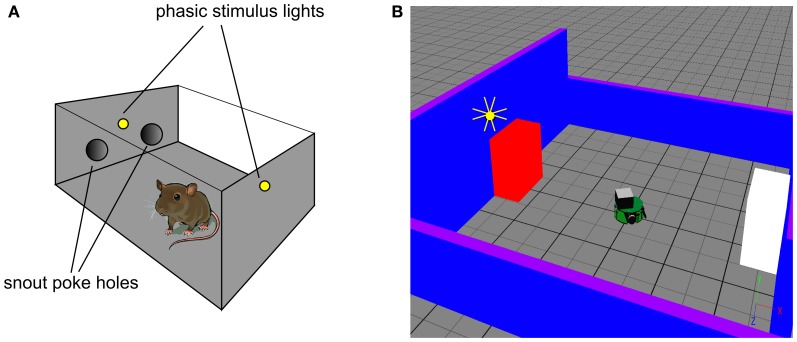
***In vivo* experimental paradigm of Gancarz et al. (2011) (panel A) and our embodied in silico counterpart (panel B). (A)** Shows the small test chamber used with rats undergoing instrumental learning. One side of the chamber has two poke holes with a light above them. Rat snout entry into the “active” poke hole may cause the two lights to flash and the active hole may be either one (for a particular rat). **(B)** Shows the virtual world created as a counterpart to that in **(A)**. A simulated Khepera I robot replaces the rat, and snout holes are replaced by colored blocks. Only the red block is ever designated the active one, and the white block corresponds to the inactive poke hole. There is a point-light located at the top of the red block which may flash if the robot bumps into the red block.

The robot task mimics an *in vivo* counterpart (Gancarz et al., 2011) in which rats spontaneously poke their snouts into one of two poke-holes in a small operant chamber (Figure 2A). Each experiment was conducted over 16 days with the rat exposed to a single 30 min session in the operant chamber each day. Critically, the animals were not food or liquid deprived, and were therefore not motivated by any extrinsic reward. The ambient light condition was complete darkness, and the rats were free to move around the chamber. In a first *habituation phase* (the first 6 days), there were no consequences to the animal making a snout entry into either poke hole. In a second *response contingent phase* (subsequent 10 days) one of the snout holes was designated the “active hole” and a snout entry here could cause a phasic light stimulus to flash briefly (mechanistically, this was achieved with two lights, one near the snout holes and one at the back of the chamber). This light flash was the only source of behavioral reinforcement and its occurrence was under control of a variable interval (VI) schedule with mean of 2 min. That is, there was a random interval (with mean 2 min) between potential snout-entry/light-flash pairings; premature snout entry into the active hole before completion of this interval caused no light flash. Snout entry into the active hole was designated an *active response* (with or without any consequent light flash) and entry into the other hole, an *inactive response*. The labeling of the snout holes in the response contingent phase is carried across to the habituation phase, although here it constitutes an arbitrary distinction. Thus, “active responses” in the habituation phase are simply those responses directed to the snout hole which becomes active during response contingency.

**Figure 3:**
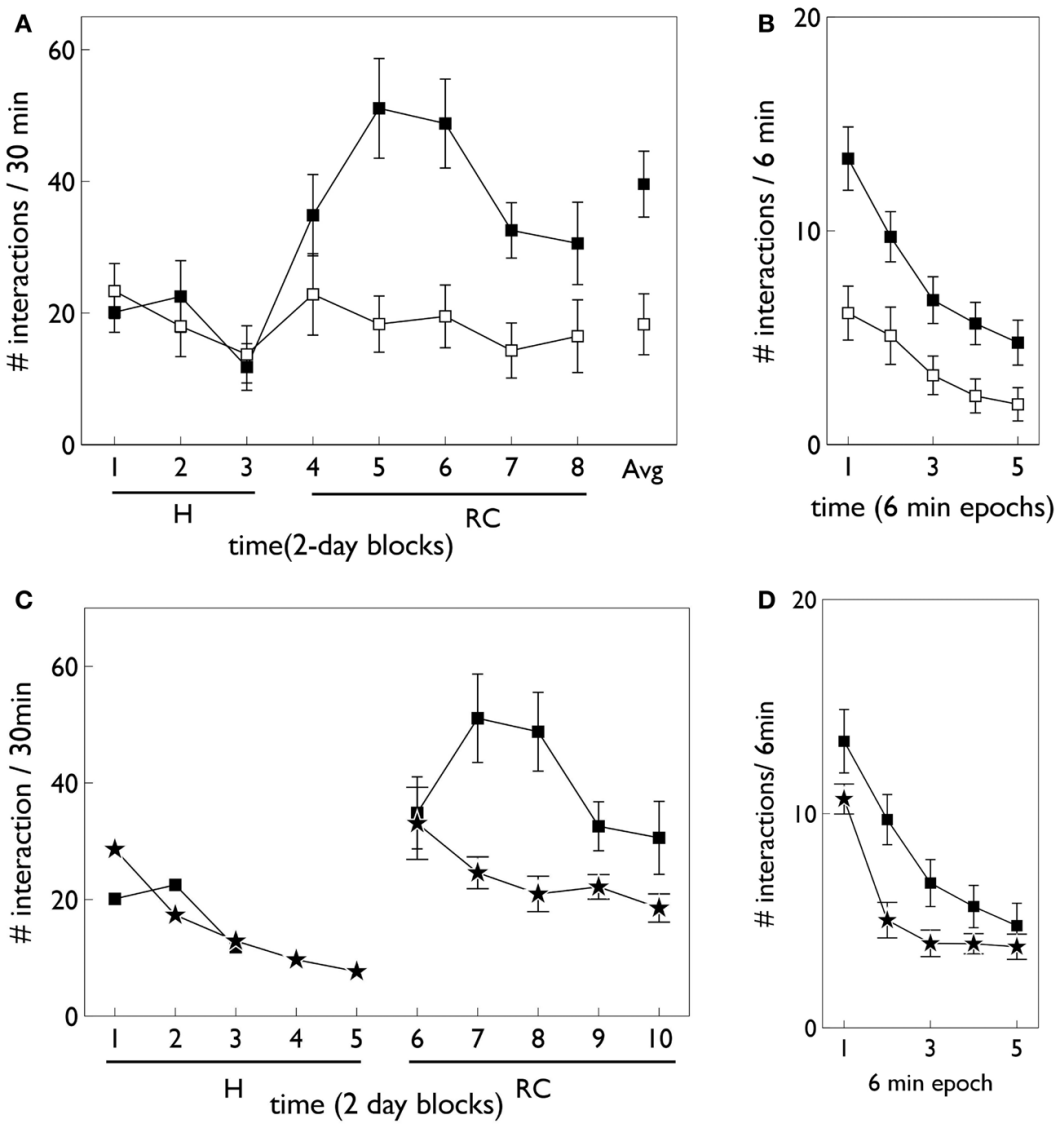
**Behavioral data adapted from the *in vivo* studies of Gancarz et al. (2011) (study 1) and Lloyd et al. (2012) (study 2). (A,B)** For variable interval (VI) training from study 1. **(A)** Shows the number of inactive and active responses in each 2-day period (averaged over the two 30 min sessions therein) with white and black symbols, respectively. The habituation and response contingent phases (see text) are designated “H” and “RC,” respectively, and the average response during the response contingent phase is shown on the extreme right as “Avg.” **(B)** Shows the within-session behavior during the response contingent phase. Results are averaged over all 10 days of this phase and means are reported for each epoch of 6 min duration during the 30 min sessions. Error bars in both panels are the mean of the standard errors for the low and high responding animals (as originally reported in study 1). **(C)** Shows active responses (star-shaped data points) from a fixed-ratio (FR1) schedule reported in study 2. Also shown for comparison are the active response in **(A)** (black squares). Note, there were more days in the habituation phase of study 2, and error bars in the habituation phase are not shown. **(D)** Is a counterpart to **(B)** with FR1 data shown by stars, and the VI data from **(B)**, replicated for comparison (black squares).

Relevant results of this experiment are shown in Figure 3. In that experiment, animals were divided in “low and high responders” according to a pre-experimental assay of overall levels of motoric activity (Gancarz et al., 2011). Here, we have averaged the data across the two groups. Figure 3A shows that there is no significant difference in responding to the two snout holes during the habituation phase. However, there is a clear difference during the response contingent phase; the animals spent more time engaging with the active snout hole. Other trends indicated are a gradual development, and subsequent decline, in the preference for the active hole during the response contingent phase. Figure 3B shows the mean behavior with each session during the response continent phase. There is a clear initial high number of active and inactive responses, and a subsequent decline in both during the session.

#### 2.1.1. Fixed-ratio variant

While the VI schedule provides valuable data to constrain the model, the action discovery paradigm, as encountered ethologically, is likely to be governed by less random reinforcement. In particular, if reinforcement is reliably given at every successful interaction with the target object, we have *fixed-ratio* (FR) schedule with ratio one (FR1). We therefore also ran simulations with this schedule.

At the time of completing this work, the corresponding biological data was not yet available and so the behavioral outcomes of the simulated agent became predictions for a similar *in vivo* experiment. However, during revision of this paper, we became aware that the laboratory responsible for the study described above had just published a followup which used an FR1 schedule (Lloyd et al., 2012). Our predictions were therefore immediately put to the test. The relevant data for the FR1 schedule from the study in (Lloyd et al., 2012) are shown in Figures 3C,D. Only active responses are shown in order to facilitate a comparison with the VI data described above (inactive responses are similar to that for the VI case). For FR1 training, the peak number of responses in the response contingent phase occurs in the first day of that phase, and shows a rapid decline thereafter (Figure 3C). In contrast, the peak response for VI training occurs after the first day of response contingency and shows a more gradual decline. Within a session, the FR1 schedule shows a steeper decline than its VI counterpart (Figure 3D).

### 2.2. Simulated robot world

We used simulation of a small autonomous robot in an arena with stimulus objects to mimic the *in vivo* experiment of Gancarz et al. (2011)—see Figure 2B. The robot was the K-Team Khepera (Mondada et al., 1999) and simulation used the Webots (v6.3.2) software environment (Cyberbotics, 2010a,b). The arena consisted of a tiled ground-plane (60 cm × 60 cm) with blue walls (two each of 10 cm and 20 cm height). The stimuli comprised two static blocks (5.9 cm by 9.8 cm by 10 cm) colored red and white, that played the role of the poke holes. Unlike the snout holes in the experiment with rats, the blocks were spatially well separated (opposite sides of the arena). For the rats, their use of local tactile (whisker-based), rather than wide-field visual information, means that the snout holes are well separated in the sensory space of the animal. This is what we achieve in the visual modality using the arrangement in Figure 2B. A light source that can flash briefly was located above the red block (there was no need for additional, rear-mounted lighting to cause sensor response in the Khepera). This light is triggered by the robot bumping into the red block (albeit possibly under VI-schedule control). The red block is therefore a surrogate for the active snout hole in the *in vivo* experiment of Gancarz et al. (2011).

The robot has a cylindrical body shape with height 3 cm and diameter 5.6 cm. Each wheel can be separately controlled to go forwards or backwards. There are eight infrared sensors in a radial configuration that were used for proximity detection in an “exploratory” behavior which also required avoiding contact with objects. The two front sensors were also used to detect the light flash. We used an RGB camera with 64(wide) × 1(high) pixel array mounted on top of the Khepera's central body to detect the colored blocks, and a binary tactile sensor at the front to detect bumping into objects. The supplementary material contains a short video showing the actions available to the virtual robot.

### 2.3. The virtual robot control architecture: overview

**Figure 4:**
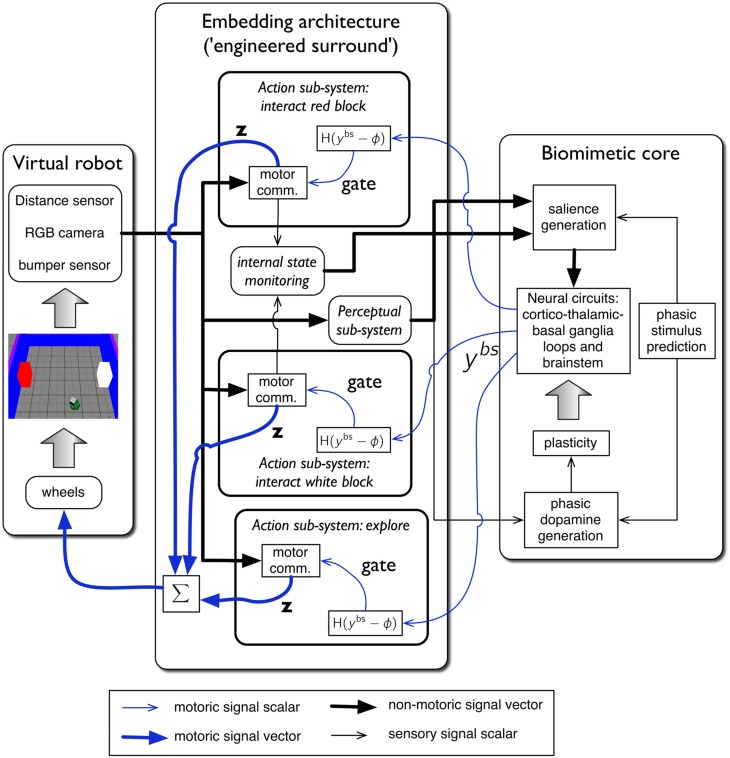
**The virtual robot control architecture, and its interaction with the robot and environment**. The virtual Khepera robot is endowed a range of sensors and the motor output is locomotion via a pair of wheels. The architecture is split into embedding, and biomimetic core, components. The embedding architecture contains three action-subsystems: two for approaching-and-bumping into each of the red and white blocks (“interact red block,” “interact white block”), and one (“explore”) for randomly roaming the arena while avoiding object contact. Within each action subsystem the motor command units are designated “motor comm.” The biomimetic core contains a biologically plausible circuit (representing basal ganglia, and its connectivity with cortex, thalamus, and brainstem), a phasic stimulus prediction mechanism, a source of phasic dopamine, and the new learning rules for basal ganglia plasticity. Other symbols and components are labeled as in the main text.

The complete virtual embedded robot model is shown in Figure 4. It comprises three principal components: the *virtual robot*—referencing its hardware, motor plant and peripheral sensors; an *embedding architecture*, or *engineered surround*, and the *biomimetic core* model. This partitioning scheme has been described in our previous work (Prescott et al., 2006; Gurney, 2009; Gurney and Humphries, 2012). The idea is to separate off the biomimetic model which is the primary subject of study, from less biologically realistic, and somewhat “engineered” components which are, nevertheless, required to produce a complete, behaving agent. In this way, we package together those elements of the architecture which are part of the model proper, and which encapsulate our hypotheses about brain function, and separate them from elements which are predicated on our hypothesis set. Thus, if we identify the cause of deficiencies in behavioral outcome with issues in the embedding architecture, we can be sure we are not falsifying hypotheses embodied in the biomimetic core. It is not necessary for a part of the biomimetic core to be a neural network; algorithmic elements are also candidates if they implement key model functions.

The key for this approach to work is the signal interface between surround and core. Thus, just as in modular software, the signals must have the same interpretation for both components either side of this interface. In our context, the embedding architecture must supply signals to a “sensory cortical” area in the biomimetic core that can interpret them as saliences for action requests, as well as any internal state variables required to modulate them. Sensory indication of phasic events must be made available to the dopamine system, and the motoric output of the biomimetic core must comprise a “selection signal” that can be used to gate actions. This signal interface is precisely that shown in Figure 4. We now go on to describe each major system in more detail.

### 2.4. Embedding architecture

The embedding architecture is based on that described by Prescott et al. (2006). The agent is supposed to have a fixed repertoire of behaviors or action-sequences, and the enactment of each one is encapsulated in an action subsystem. In the current model there are three such behaviors:

**Explore:** move around the arena and avoid obstacles (blocks and walls).
**Interact with the red block:** orient to the red block, approach it, and perform a controlled “bump” into it. This latter comprises, in turn, the following sub-actions: bump once against the red block, move backwards, stop, and then slowly approach the red block again.
**Interact with the white block:** this is identical to its counterpart for the red block, except actions are directed to the white cube.

The block-interaction behaviors are surrogates for the snout hole poking in the *in vivo* experiment of Gancarz et al. (2011). The key difference in outcome between the two behaviors is that interaction with the red block causes the light flash—it comprises the active response—whereas interaction with the white block has no consequences and comprises the inactive response.

The granularity of behavior encoded in each action sub-system is clearly quite coarse; we have already noted that they each comprise small action sequences. Thus, they have similarities with the *fixed action patterns* (FAPs) of the ethologists (Lorenz, 1935; Tinbergen, 1951) and the *options* used in hierarchical RL (Barto et al., 2004). This is not a drawback in the current model as we are primarily interested in the basic principles of adaptive aspects of behavior with novel stimuli, and any consequent plasticity; the precise semantics of each action are not important. Further, the behaviors we encode are not as rigid as FAPs or options, as our method of behavioral maintenance—an excitatory recurrent connection within the motor cortex (see “Appendix”)—allows the behaviors to be interrupted by “exploration” if this has sufficiently high salience. We will revisit the issues surrounding action granularity in the section 4.

Within each action-subsystem, the sequencing of primitive actions into behaviors is accomplished in a *motor command unit*. These units make use of sensory information to trigger various events in the sequence. The “explore” behavior is governed by the infra-red sensors which detect distance to objects in the robot's path, thereby allowing locomotion while avoiding objects. The block-interaction behaviors use camera information to identify, and orient to the blocks, and the bumper sensor to know when contact has been made.

The motor output of each motor command unit is 2-vector **z** = (*z*_*l*_, *z*_*r*_) whose components indicate the desired speed for each robot wheel (left and right) to enact the current segment of behavior. The motor command units are not neural networks but conventional procedural code which use sensor information to trigger the next action component in the sequence, and update **z** at each time step. If the behavior in the action subsystem has been selected by the biomimetic core, then the corresponding speed-output vector is sent forward to be averaged with output vectors from any other selected sub-systems. In this way, multiple selected actions are blended together to produce a final behavior. This forces a strong test of the action selection capability of the biomimetic core model which must prevent over-expression of such multiple action selection.

The selection criterion for an action subsystem *i*, is that the corresponding brainstem output signal from the biomimetic core, *y*^*bs*^_*i*_ should exceed some threshold ϕ. That is, *H*(*y*^*bs*^_*i*_ − ϕ) = 1, where *H*() is the Heaviside function. In our simulations ϕ = 0.5.

The *perceptual sub-system* supplies sensory information for generation of the salience of the action requests for the block interaction behaviors. In the first instance, this is quite simple; the perceptual subsystem detects the presence of the red/white block in the visual field and triggers a salience for the red/white block-interaction behavior. However, the salience of the blocks is subject to a variety of additional processes driven by sensory habituation and perceived novelty of the stimulus. These processes are based on biological notions and so we reserve them for the biomimetic core. They also depend on the status of the block-interaction behaviors (completion of a block interaction cause an habituation increment). Therefore, these two command units also provide signals to an *internal state monitoring* unit that indicate if their respective sequences have recently been completed. This unit also provides a representation of the motivation to explore the arena, governing the selection of the “explore” action sub-system. Finally, the perceptual subsystem also provides a signal to the dopamine system about phasic events such as the light flash.

### 2.5. The biomimetic core

The biomimetic core comprises several functional blocks (see Figure 4)—we now deal with each in turn.

#### 2.5.1. Prediction of phasic stimuli

A key component in our model is the idea that the phasic outcome of the interaction with the blocks (the light flash) is subject to prediction via an internal model. This prediction is then used to modify the salience of objects in the visual field at the time of the light flash (the blocks) and also to form a sensory prediction error which forms the basis for the phasic dopamine signal.

Prediction is believed to be a fundamental process at the heart of perception and cognition (Bar, 2007; Bubic et al., 2010; Friston, 2010; Gurney et al., 2013) and is, in general, a complex neural process requiring substantial model resources. However, formalizing a *phenomenological* model of prediction of the phasic light flash is straightforward if we assume that the latter is represented by a single scalar feature *y*_*f*_(*t*) whose value is binary: a 1 signals the detection of a light, and 0 its absence (no light flash). The prediction is then a real-valued scalar between 0 and 1, where values close to 1 or 0 are strong predictions that the light will flash on or be absent, respectively.

To proceed further, consider the set of times {*t*_*i*_} when the light flash *might* occur (during block-interactions), where *i* indexes the block-interactions over the entire (multi-day) experiment. We distinguish between the phasic *manifestation* of the prediction y^*^_f_(*t*_*i*_), at discrete time *t*_*i*_, and the internal *latent* representation of the prediction *y*^(*)^_*f*_(*t*) which exists at all times *t*. The phasic prediction is supposed to correspond to phasic neural activity, whereas its latent counterpart is encoded in the structure (synaptic weights) of the internal model of prediction.

The model we use is phenomenological and we use a similar approach, based on exponential rise and decay as is used with habituation (Marsland, 2009). Thus, if a phasic event (light flash) occurs at *t*_*i*_, the prediction is increased according to the recursive relation

(1) $$y_{f}^{\left(*\right)}\left(t_{i}+\delta t\right)=1-k\left(1-y_{f}^{\left(*\right)}\left(t_{i}\right)\right)\;where\;0<k<1$$

**Figure 5:**
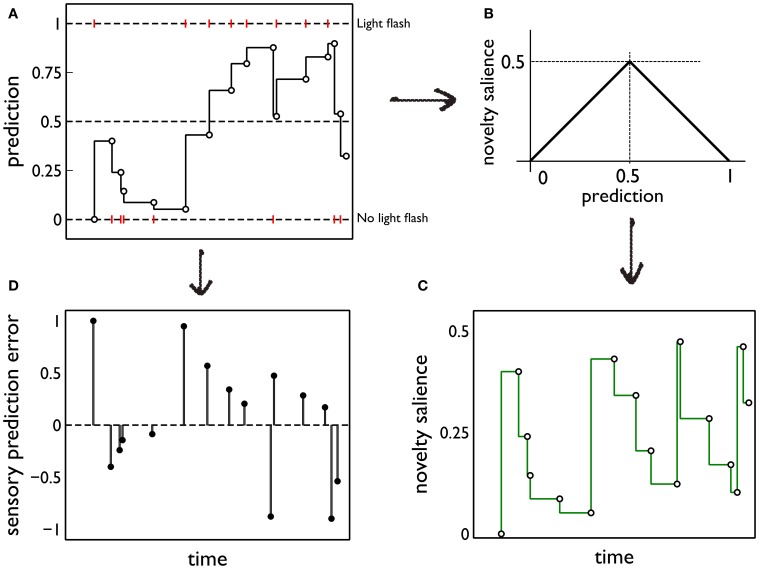
**Prediction and its deployment for novelty salience and sensory prediction error under a simple phenomenological model. (A)** The red markers indicate the presence or absence of phasic outcome (light flash) during each interaction with the red (active) block. The latent prediction, *y*^(*)^_*f*_(*t*), is shown as the solid line and the phasic prediction, y^*^_f_(*t*_*i*_), by the open markers. **(B)** The translation of prediction into novelty salience. **(C)** The time course of novelty salience corresponding to the prediction in **(A)**, obtained via the mapping in **(B)**. Open circles represent the salience perceived at each block interaction, when the block is in view. These bouts of block-perception are longer than the observation of the light flash, but we identify each interaction with a point-time marker for simplicity. The continuous line is a formal mapping of the latent prediction using Equation (3). **(D)** The sensory error signal derived from **(A)**.

This occurs within days and across day boundaries, because we assume no day-to-day unlearning of the internal model for prediction of phasic outcome. The definition is completed by defining the effect of the first reinforcing event: *y*^(*)^_*f*_(*t*_1_ + δ*t*) = 0.2. If, after a block-interaction, there is a non-zero prediction of a phasic outcome which was not delivered (no light flash), then the prediction is updated according to

(2) $$y_{f}^{\left(*\right)}(t_{i}+\delta t)=ky_{f}^{\left(*\right)}(t_{i})$$

(both within and between days). Thus, latent prediction is constant for the intervals *t*_*i*_ < *t* ≤ *t*_*i* + 1_. Then, when activated by sensory cues, the model delivers the phasic prediction y^*^_f_(*t*_*i*_) = *y*^(*)^_*f*_(*t*_*i*_). For all our simulations, *k* = 0.95. Figure 5A shows a cartoon of a typical sequence of events and the resulting predictions.

#### 2.5.2. Salience generation

Salience for the block interaction behaviors is initiated by the perceptual subsystem being activated by the presence of a colored block in the field of view. This generates a nominal salience value which is then subject to habituation, dishabituation, and possibly a sensitization due to novelty. We refer to the nominal salience of the colored blocks modulated by (dis)habituation as the *intrinsic* salience of the blocks. This may be augmented by a separate *novelty salience*; both contributions are detailed below.

Habituation is defined as “a behavioral response decrement that results from repeated stimulation and that does not involve sensory adaptation/sensory fatigue or motor fatigue” (Rankin et al., 2009). Evidence for habituation in the *in vivo* experiment of Gancarz et al. (2011) comes from close examination of the data in Figure 3. There is clear evidence of a decline of inactive responses within each session (day) of the response contingent phase. There is also some indication of similar trends across days with in each phase of the experiment. Thus, linear fits to the means of inactive responses have a negative slope within each phase and, for the habituation phase, this was a significant trend (Gancarz et al., 2011). The inactive responses are least likely to be subject to any contribution from novelty and represent (as far as possible) a control stimulus. We therefore assume any behavioral changes in inactive responses are a consequence of the dynamics of the intrinsic salience of the stimuli. Thus, we incorporated salience habituation processes, both across, and within days, resulting in the decline of the intrinsic salience of both blocks on these two time scales.

It might be thought that the decline within a session could be due to a general “fatigue.” However, this can be ruled out for several reasons. First, there is little effort in a snout poke response, and it is part of the normal behavioral repertoire of the rat. Second, there is ample use in behavioral studies of testing rats for much longer than the 30 min sessions used here. Third, in the study by Lloyd et al. (2012), animals confronted with a more difficult (VI) learning schedule, showed more responses within a session than those under a less demanding, fixed-ratio schedule. We therefore conclude that decrements in response are due to genuine adaptive neural processes.

Habituation is usually accompanied by a dishabituation process whereby, presentation of alternative stimuli, or a “rest period,” allows habituated behavioral responses to recover to previously observed levels (Groves and Thompson, 1970; Rankin et al., 2009). These complementary processes may be modeled using simple exponential forms (Marsland, 2009), and we used this general approach in the following way. Thus, let *S*^*i*, *j*^_int_ be the intrinsic salience *during* the *j*th block-interaction on day *i*, given the associated block is in the visual field. Within a session, we do not update salience from moment to moment, but rather after each complete interaction with the block. This is consistent with recent ideas about habituation that include reference to response rate change in operant tasks (McSweeney and Murphy, 2009; Rankin et al., 2009). Therefore at the start of the (*j* + 1)th interaction, *S*^*i*, *j* + 1^_int_ = γ_*b*_ *S*^*i*, *j*^_int_, with γ_*b*_ < 1. At the start of the next day, there is a re-initialization *S*^*i* + 1, 1^_int_ = γ_*a*_ *S*^*i*, 1^_int_, where γ_*a*_ < 1. Typically, as a result of this, there is dishabituation between days (so that, if $\hat{j}$ is the last interaction on day *i*, $S_{\text{int}}^{i,\;\hat{j}}<S_{\text{int}}^{i+1,\;1}$). Parameters were *S*^1, 1^_int_ = 0.45, γ_*a*_ = γ_*b*_ = 0.95.

We now suppose there may be an additional salience contribution to the target block interaction associated with the surprising phasic outcome (light flash). Thus, we make the hypothesis that objects or features in the perceptual field when a surprising phasic event occurs, acquire *novelty salience* by a process of “inheritance” or generalization from the surprise of the simple phasic outcome (e.g., light). This is an extension to neutral stimuli of the observation that sensitization usually occurs during the first few presentations of a (non-neutral) rewarding stimulus (McSweeney and Murphy, 2009). It is also consistent with the fact that habituation (the counterpart of sensitization) can engender generalization to other stimuli (Rankin et al., 2009).

To quantify this idea we assume that the novelty salience is maximum when the outcome of the interaction is least predictable or most uncertain; that is, when y^*^_f_ is at its intermediate value of 0.5. For, at this point, there is no bias in the prediction of the phasic stimulus occurring or being absent. We then assign a novelty salience of zero to the “firm predictions” corresponding to y^*^_f_ = ± 1, and assume piecewise linearity elsewhere. This mapping is shown in Figure 5B. Formally, if *S*^*i*, *j*^_nov_ is the novelty salience for interaction *j* on day *i*, at time *t*_*i*, *j*_,

(3) $$S_{\text{nov}}^{i,j}=0.5-\left|y_{f}^{*}\left(t_{i,j}\right)-0.5\right|$$

The ensuing novelty salience from the events in Figure 5A is shown in Figure 5C. The total salience is given by

(4) $$S_{\text{tot}}^{i,j}=S_{\text{int}}^{i,j}+S_{\text{nov}}^{i,j}$$

Salience only occurs when the stimuli are perceived (at the points indicated by the open circles in Figure 5C). However, it is useful to indicate the causality of changes in novelty salience by formally transforming the latent prediction using Equation (3) so into novelty salience after each interaction is the salience that *would* be seen if the stimulus comes into view.

The salience for the exploratory action is assumed to be driven by an internal motivational process (like fear or foraging for food) which is notionally a component of “internal state monitoring.” It manifests itself in a salience for exploration drawn from a uniform distribution with constant mean of 0.4, and standard deviation of 0.23.

#### 2.5.3. Basal ganglia and loops through cortex

**Figure 6:**
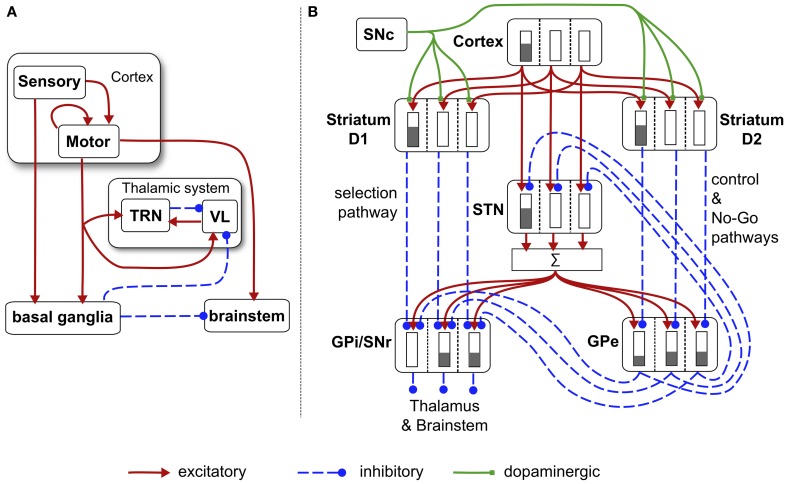
**Schematic diagram of the basal ganglia neural network component of the biomimetic core. (A)** Cortex, basal ganglia, brainstem, and thalamic complex. The latter is comprised of the thalamic reticular nucleus (TRN) and ventrolateral thalamus (VL). Note that action channels are present but not explicitly shown here. **(B)** The basal ganglia circuit consisting of: striatal projection neurons expressing D1 or D2 dopamine receptors; subthalamic nucleus (STN); output nuclei—globus pallidus internal segment (GPi) and substantia nigra pars reticulata (SNr); globus pallidus external segment (GPe), and substantia nigra pars compacta (SNc). The three action channels are shown in this panel, and a typical set of activities indicated in cartoon form by the gray bars (the channel on the left is highly salient causing suppression of basal ganglia output on that channel). The summation box below STN is not anatomically present—it is graphic device to indicate that each target of STN sums its inputs across channels from STN.

The main neural circuit in the biomimetic core is based on our previous work with models of basal ganglia (Gurney et al., 2001a,b) and loops through cortex (Humphries and Gurney, 2002). Key concepts were outlined in the Introduction; details of the particular form used here are shown in Figure 6. The model uses discrete processing channels for each action so that, within each nucleus, there is a localist representation of each channel as a population of neurons instantiated in a leaky integrator neural unit. Formally, each neural unit has an activation variable *a* governed by a first order ODE

(5) $$\tau \frac{da}{dt}=-a\left(t\right)+I\left(t\right)$$

where τ is the characteristic membrane time constant (here, τ = 40 ms) and *I* is the summed, weighted input. The normalized firing rate *y*, of the neural unit is given by a piecewise linear squashing function

(6) $$y(a)=L(a,\;\epsilon )=\{\begin{array}{ll} 0 & a\leq \epsilon \\ a-\epsilon & \epsilon <a<1+\epsilon \\ 1 & a>1+\epsilon \end{array}$$

where, ϵ is a threshold below which *y* = 0, immediately above which *y* depends linearly on *a* with unit slope, and there is saturation at *y* = 1.

There are three channels in the current model—one for each of the action-subsystems. The sensory cortex (Figure 6A) receives input from the salience generators, and initiates activity in motor cortex. This activity can potentially undergo amplification in the recurrent loop with the thalamic system, but this is under basal ganglia control. The motor cortex and the basal ganglia output nuclei project directly to the reticular formation and pendunculopontine nucleus brainstem areas a (Takakusaki et al., 2004; Jenkinson et al., 2009). If the increased drive from motor cortical channel *i* to its corresponding brainstem population, as well as the direct release of inhibition from that population, cause its activity *y*^*bs*^_*i*_ to exceed the threshold ϕ, then the channel is selected for behavioral expression (see Figure 4).

Within the basal ganglia, there are two interdigitated populations of projection neurons in the main input nucleus—the striatum. These so-called *MSNs* are differentiated according to their preferential expression of dopamine receptor type—D1 or D2. We refer henceforth to these populations as *D1-striatum* and *D2-striatum*. The subthalamic nucleus (STN) is the only source of excitation in basal ganglia. The output nuclei of the basal ganglia are the globus pallidus internal segment (GPi) and substantia nigra pars reticulata (SNr). The circuit comprising D1-striatum, STN and GPi/SNr form a feedforward, off-center, on surround network implementing an inter-channel competition; hence it is dubbed the *selection pathway*. The “winning” channel in basal ganglia competitive processes is that which has the lowest output in GPi/SNr (inhibition to targets is released). This channel will have received the largest inhibitory input fron D1-striatum, which, in turn, will have been subject to the highest salience input. The circuit comprising the globus pallidus external segment (GPe), STN and D2-striatum exercise a *control* function acting on the selection pathway to ensure a good match between overall excitation from STN, and striatal inhibition of the output nuclei (Gurney et al., 2001a,b). The circuit through D2-striatum, GPe and SNr also implements a NO–GO function, actively preventing action selection (Frank et al., 2004). Parametric details of the application of Eqs. (5) and (6) to the circuits in Figure 6 are given in the “Appendix.”

The cortico-striatal synapses receive modulatory input from dopamine axons which branch profusely throughout striatum (Beckstead et al., 1979; Gauthier et al., 1999; Matsuda et al., 2009). Dopamine terminals also seem to innervate striatum in a dense, non-focal way within the neuropil of striatum (Moss and Bolam, 2008), and dopamine also acts extra-synaptically via volume transmission (Cragg and Rice, 2004). These data would indicate a diffuse innervation of striatum by dopamine neurons that cuts across channel boundaries.

Tonic (background) dopamine levels are thought to influence cortico-striatal transmission at D1 and D2 MSNs in opposite ways with D1/D2 receptors facilitating/attenuating cortico-striatal transmission (West and Grace, 2002). This is incorporated into our model by including a constant tonic dopamine level λ, which increases cortico-striatal D1-MSN weights by a multiplicative factor 1 + λ, and decreases corresponding D2-MSN weights by 1 − λ. More significantly for the current study are the dynamics of phasic (transient) dopamine, which are critical for cortico-striatal plasticity (Reynolds and Wickens, 2002), and to which we now turn.

#### 2.5.4. Phasic dopamine and sensory prediction error

The starting point for this component of the model is our hypothesis that phasic dopamine signals a sensory prediction error (Redgrave and Gurney, 2006; Redgrave et al., 2008). Using the notation developed in section 2.5.1, the sensory prediction error *e*(*t*_*i*_) is given by *e*(*t*_*i*_) = *y*_*f*_(*t*_*i*_) − y^*^_f_(*t*_*i*_). The error resulting from the sequence of events in Figure 5A is shown in Figure 5D. In the rest of this section, we drop the temporal argument and its indexing as it assumes a single block interaction.

However, we also wish to relate this form for *e* to its biological generation and realization in phasic dopamine. In particular, we invoke the evidence that phasic dopamine is released in response to neutral phasic stimuli and that this occurs via the recently discovered tecto-nigral pathway (Coizet et al., 2003; Comoli et al., 2003; Dommett et al., 2005). This is a direct (mono-synaptic) pathway between the superior colliculus (SC) (optic tectum in non-mammals) and midbrain dopamine neurons in substantia nigra pars compacta (SNc). The SC plays a key role in gaze shifting and orienting responses (Wurtz and Goldberg, 1972; Wurtz and Albano, 1980) and is believed to act as a detector of novel, phasic stimuli (Dean et al., 1989). In our terminology it detects *y*_*f*_. Phasic responses in SC then excite SNc neurons and therefore potentially cause phasic bursts of activity therein. However, as the stimulus becomes predictable, this response in SNc disappears and, significantly, if the predicted reward is omitted, there is a phasic “dip” in the dopamine response below tonic level (Schultz et al., 1997; Schultz, 2006). Taking these pieces of evidence together, suggest that the null response in SNc under stimulus prediction is a result of the excitatory influence of SC, and a similarly timed inhibitory signal from another nucleus which we will call the “canceling signal.” The lateral habenula may be a candidate for such signals in dopamine neurons (Matsumoto and Hikosaka, 2007).

To model the SC, we assume that its response is not only contingent on *y*_*f*_ but also on any phasic prediction y^*^_f_. This extends the temporally adaptive response of colliculus at long time scales under habituation (Drager and Hubel, 1975) to include phasic prediction at shorter time scales. Thus, if *y*^*SC*^_*f*_ is the response of SC to phasic feature *f*, we put *y*^*SC*^_*f*_ = [*y*_*f*_ − y^*^_f_]^+^, where [*x*]^+^ = max(0, *x*). Then, the canceling signal *y*^*C*^_*f*_ takes the form *y*^*C*^_*f*_ = [y^*^_f_ − *y*_*f*_]^+^ and the sensory prediction error is given by

(7) $$e=y_{f}^{\text{SC}}-y_{f}^{\text{C}}={\left[y_{f}-y_{f}^{*}\right]}^{+}-{\left[y_{f}^{*}-y_{f}\right]}^{+}=y_{f}-y_{f}^{*}$$

Since the collicular and canceling signals are not derived from prior inputs, we modeled their dynamics phenomenologically so that each of *y*^*SC*^_*f*_, *y*^*C*^_*f*_ are triangular pulses of width 0.2 s.

In translating this into dopamine activity in our model there are several issues to contend with. First, we don't know the relation between positive and negative excursions of *e* and phasic dopamine bursts and dips—it could be that an error of +1 is signalled by a dopamine level many times that of tonic, but that an error of −1 is signalled by sufficiently prolonged dip with minimum of zero. We are therefore free to include parameters *a*^+^, *a*^−^ in forming the effective input to a dopamine neuron, *I*^SNc^, which encodes prediction error

(8) $$I^{\text{SNc}}=a^{+}y_{f}^{\text{SC}}-a^{-}y_{f}^{\text{C}}$$

These parameters were chosen for best model fit to the data of Gancarz et al. (2011) giving *a*^+^ = 2, *a*^−^ = 1. Further, we don't know *a priori* the relationship between the magnitude of *e* (which lies in the interval [−1, 1]) and the corresponding level of simulated dopamine, *d*, expressed in our plasticity rules. We therefore use *I*^SNc^, to determine an *effective* SNc output, *y*^SNc^, which we can then equate with *d*. Thus, we form the SNc activation *a*^SNc^ in a first order ODE like that in Equation (5) and use this, in turn, to generate *y*^SNc^ ≡ *d* via the function

(9) $$y^{\text{SNc}}=\{\begin{array}{ll} 0, & a^{\text{SNc}}\leq -0.2\\ a+0.2, & a>0.2 \end{array}$$

The lack of normalization is a requirement for interpreting *y*^SNc^ as the simulated dopamine level *d*, used in the next section.

#### 2.5.5. Cortico-striatal plasticity: the learning rule

The learning rule is based on our recent work on cortico-striatal plasticity at the level of spikes (Gurney et al., 2009) which is, in turn, grounded in a comprehensive *in vitro* study (Shen et al., 2008). The latter was able to distinguish recordings between D1 and D2-type MSNs, and yielded responses at different levels of dopamine. The resulting learning rules are complex and reflect the unavoidable complexity in the data. However, the rules do provide an account of plasticity consistent with action discovery and so we sought to incorporate them in the current model. Fortunately Pfister and Gerstner (2006) have shown how to relate spike timing dependent plasticity (STDP) to the Bienenstock, Cooper, and Munro (BCM) rule for rate-coded neurons (Bienenstock et al., 1982; Cooper et al., 2004) which therefore allows us to proceed with this programme.

The work of Pfister and Gerstner (2006) dealt with STDP for spike pairs and triplets. The transition to firing rates is done by calculating the expected weight change 〈*dw*/*dt*〉. Let the pre- and post-synaptic firing rates be *x* and *y*, respectively. If Δ*t* = *t*_post_ − *t*_pre_ is the time interval between post- and pre-synaptic spike pairs then let τ^+^, τ^−^ be time constants associated with processes for Δ*t* > 0, Δ*t* < 0, respectively. The rate coded rule takes the form

(10) $$\begin{array}{l} \langle \frac{dw}{dt}\rangle =A_{3}\tau^{+}\tau^{y}y(y-\theta_{\text{BCM}})x\\ \;\theta_{\text{BCM}}=\langle y^{2}\rangle C_{\text{BCM}}\\ \;C_{\text{BCM}}=\frac{-(A^{-}\tau^{-}+A^{+}\tau^{+})}{A_{3}\tau^{+}\tau^{y}} \end{array}$$

Here, τ^*y*^ is a time constant associated with spike triplets, and *A*_3_ is a factor for the plasticity from triplet timing. This has no direct counterpart in our spiking level model but we assume a positive value.

**Figure 7:**
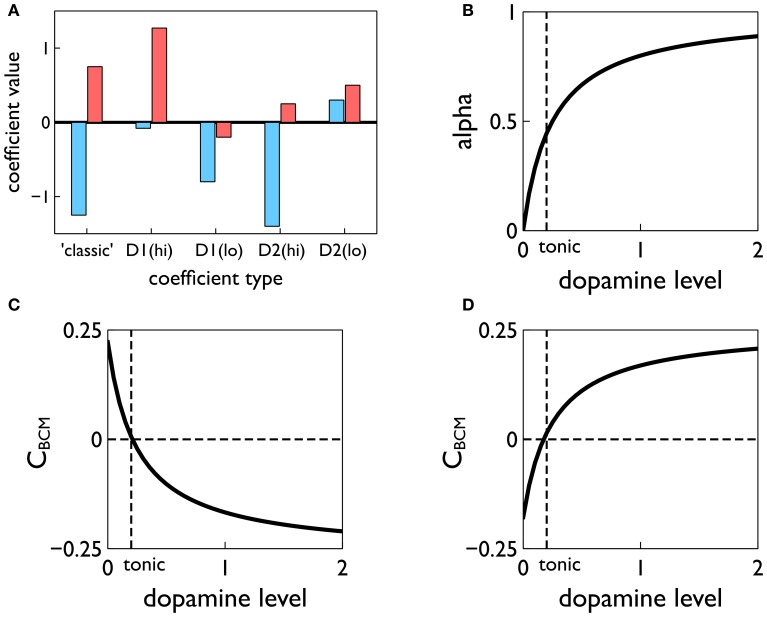
**Construction of the learning rule. (A)** The plasticity coefficients consistent with the data of Shen et al. (2008). **(B)** The dopamine mixing function α(*d*) defined in Equation (11). **(C,D)** The dopamine-dependent versions of the factors *C*_BCM_ in Equation (10) for D1 and D2-MSNs, respectively.

More importantly, the terms *A*^+^, *A*^−^ are derived from the contributions to plasticity from positive and negative spike pair timing [here they are signed quantities; in (Pfister and Gerstner, 2006) they are absolute magnitudes]. Further, we endow them with dopamine dependence and specificity under the D1/D2 MSN dichotomy. Thus, following (Gurney et al., 2009) we use the data of Shen et al. (2008) to determine these terms for D1-MSNs at high levels of dopamine *A*^D1(hi)^_+_, *A*^D1(hi)^_−_, at low levels of dopamine *A*^D1(lo)^_+_, *A*^D1(lo)^_−_, and for corresponding quantities for D2-MSNs; we refer to these eight quantities as *plasticity coefficients*. For example, with positive spike-pair timing in D1-MSNs at high levels of dopamine, the data imply strong LTP, and for negative spike-pair timing, weak LTD (Shen et al., 2008). This led to the assignment shown in Figure 7A (see “D1(hi)” bar grouping). Other coefficient assignments are shown in Figure 7A and compared with the “classic” finding for STDP in hippocampus and cortex, in which with LTP/LTD is associated with positive/negative Δ*t* (Song et al., 2000). Notice that several of the coefficient pairs give LTP/LTD assignments which are “non-classical”; for example, D2-MSNs at low dopamine have uniform LTP for both timings.

At levels of dopamine, *d*, intermediate between the “low” and “high” extremes, we define *A*^D1/D2^_±_(*d*) as a function of dopamine by “blending” the relevant plasticity coefficients together using a monotonic, saturating function α(*d*) (see Figure 7B)

(11) $$\alpha (d)=\frac{4d}{1+4d}$$

For example, for D1-MSNs, *A*^D1^_+_ is given by

(12) $$A_{+}^{\text{D1}}\left(d\right)=\alpha \left(d\right)A_{+}^{\text{D1}(\text{hi})}+\left(1-\alpha \left(d\right)\right)A_{+}^{\text{D1}(\text{lo})}$$

with similar relations for *A*^D1^_−_(*d*), *A*^D2^_+_(*d*), *A*^D2^_−_(*d*). This gives, in turn, functional forms *C*_BCM_(*d*) derived from scalar factors *C*_BCM_ in Equation (10) (see Figures 7C,D).

Weights from both motor cortex and sensory cortex to striatum (“motor weights” and “sensory weights,” respectively) are subject to the learning rule described above. The motor weights are supposed to endow the agent with the ability to perform the three actions expressed in the action-subsystems. They are initialized in such a way as to allow this to occur in the presence of the exploration action, during an initial “weight calibration” learning session. In contrast, the sensory weights are initialized to zero, and any positive increments therein are thought of as supplying new “biases” in the selection of the three given actions, derived from contextual information. However, the uniform treatment of both motor and sensory weights means their trajectories will mirror each other in form (see for example, Figure 10).

## 3. Results

### 3.1. Cortico-striatal plasticity alone is not sufficient to account for variable interval training data

**Figure 8:**
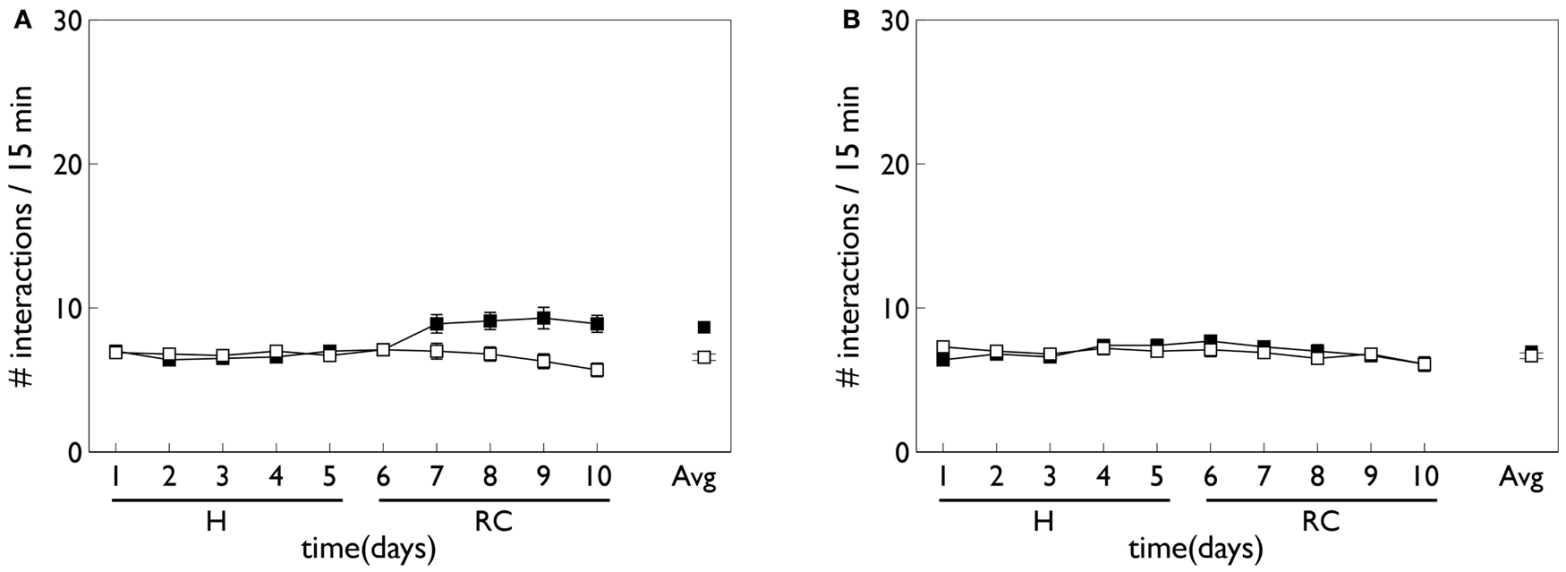
**Behavior of an agent with no novelty salience or internal prediction model, performing the block-bumping experiment. (A,B)** For models with, and without, phasic dopamine, respectively (pDA, no-pDA), and each plot is an average over 10 runs. These plots are based on those of the *in vivo* data in Figure 3. Thus, each panel shows the number of interactions with the block stimuli in each 15 min session comprising a “virtual day” of learning, plotted against such days. Error bars are 1 standard error of the mean. Open symbols are for the white (control) block while solid symbols are for the red block, which elicits a phasic outcome in the response contingent phase (labeled “RC”). The habituation phase (when there is no environmental phasic outcome) is designated “H.” The average of the interactions for each block over the entire response contingent phase is shown in the pair of data points on the extreme right of each panel.

Figure 8 shows the behavioral outcome for an agent with no novelty salience (or its associated internal prediction model), undergoing VI training in the block-bumping task. Results are averaged over 10 repetitions with different initial random number seed, and the two panels show outcomes with and without phasic dopamine enabled. This dichotomy will be a recurring theme as we wish to explore the relative contributions of novelty salience and phasic dopamine during learning. We will refer to models with and without phasic dopamine enabled as “pDA,” and “no-pDA” models, respectively. In the presence of phasic dopamine, there is a statistically significant difference between the number of interactions with the control (white) and target (red) blocks. However, this difference is nowhere near as substantial as that shown in the data of Gancarz et al. (2011). We conclude that other mechanisms must be at work and therefore invoked the notion of *novelty salience* as described in the section 2.

### 3.2. Novelty salience can account for behavioral trends in variable interval learning

**Figure 9:**
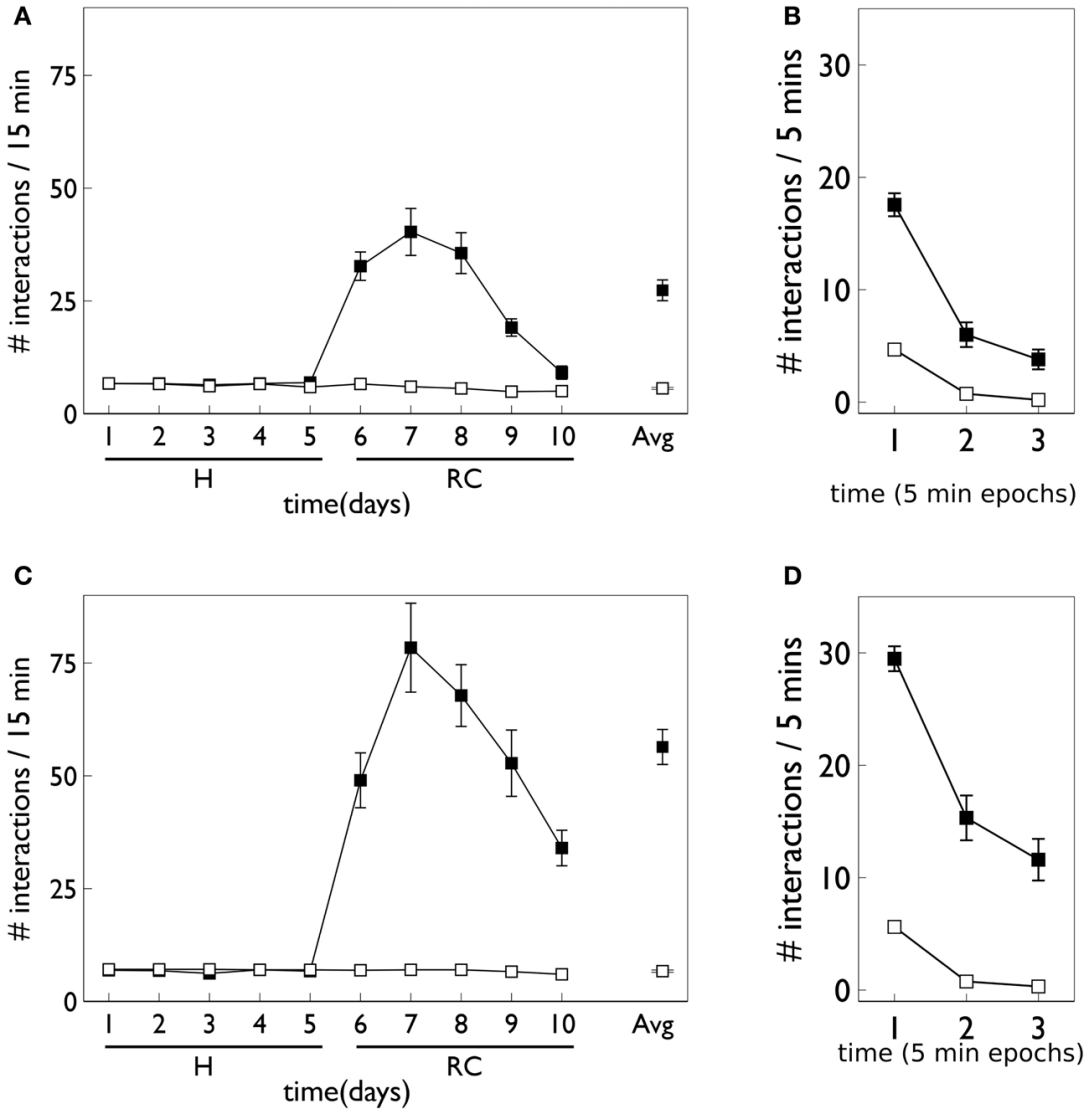
**Behavior of an agent with novelty salience and feature prediction performing the block-bumping experiment with variable interval training. (A,C)** Have a similar interpretation to counterparts in Figure 8 and are for pDA and no-pDA models, respectively. **(B,D)** Show the behavior within a virtual “day” (considered as three, 5 min epochs), averaged over the response contingent phase; **(B,D)** are for pDA and no-pDA, respectively.

Figure 9 shows the behavior for an agent in the presence of novelty salience and an internal prediction model (see section 2) undergoing VI learning (results are averaged over 10 repetitions). Both pDA and no-pDA models show qualitatively similar behavior to that from the *in vivo* experiment in Figure 3. That is, they show a substantial increase in active responses during the response contingent phase which declines toward the end of the experiment. In addition, the peak response does not occur on the first day of training in the response contingent phase. However, the no-pDA model shows markedly more active responses during the response contingent phase than its pDA counterpart. To quantify this, let *r*_peak_, be the ratio (rounded to nearest integer) of the peak number of active responses during response contingency to the mean inactive response over this time. Note that, while absolute numbers of responses in the model are not directly comparable with those *in vivo*, we might expect ratios of responses under different regimes to be more so. For the *in vivo* experiment *r*_peak_ = 3, while for pDA and no-pDA models *r*_peak_ = 7, 12, respectively. This feature is therefore more realistically captured with the inclusion of phasic dopamine.

**Figure 10:**
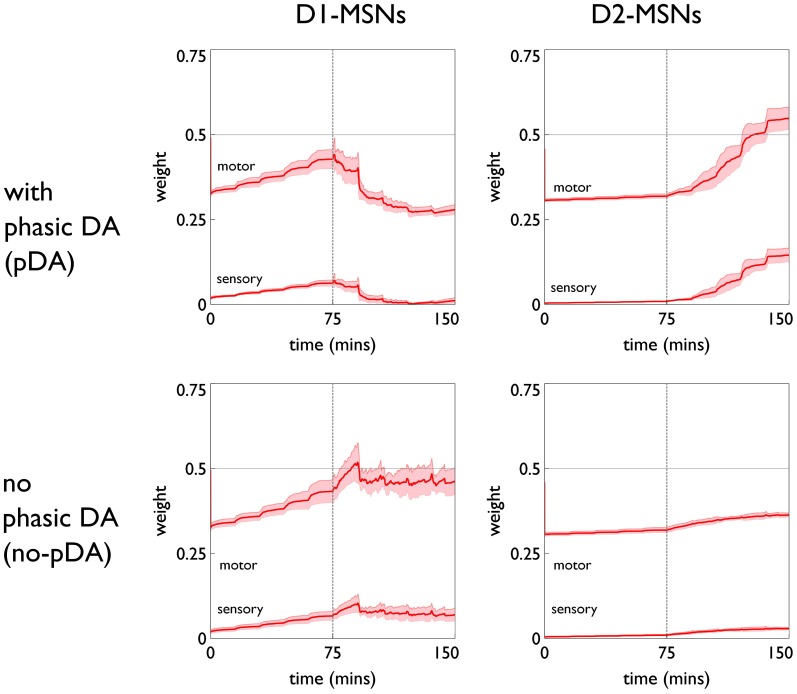
Weight trajectories w^*^(*t*) for the active response channel, in models with novelty salience and prediction, undergoing variable interval training. Rows are for pDA and no-pDA models, columns for D1- and D2-type MSNs. Weights from motor cortex and sensory cortex are labeled “motor” and “sensory.” The trajectories are expressed as continuous functions of time to show both within-day, and between-day dynamics, and the onset of the response contingent phase (at the start of day 6) is indicated at 75 min. These plots capture the statistics of the weights over a group of 10 models; the dark red line is the mean, and the red-shaded region encompasses ± 1 std dev.

The role of phasic dopamine in explaining these differences in active responses is made apparent by reference to Figure 10, which shows the dynamics of the cortico-striatal weights in the active response (red-block-interaction) channel as learning progresses. For the no-pDA model there is (unsurprisingly) little change in the weights in the response contingent phase (for both D1 and D2-MSNs, and motor and sensory cortical inputs). However, for the pDA model, there is a decrease in D1-MSN weights and an increase in D2-MSN weights. This is consistent with a decrease in the ability of the selection pathway in basal ganglia to facilitate an active response, and an increase in the potential of the NO–GO pathway to suppress it (Frank et al., 2004) (see section 2.5.3). Phasic dopamine, and the biologically plausible learning rule, are therefore directly responsible for the relative, overall difference in active responses in the pDA model, compared to its no-pDA counterpart (Figure 9).

**Figure 11:**
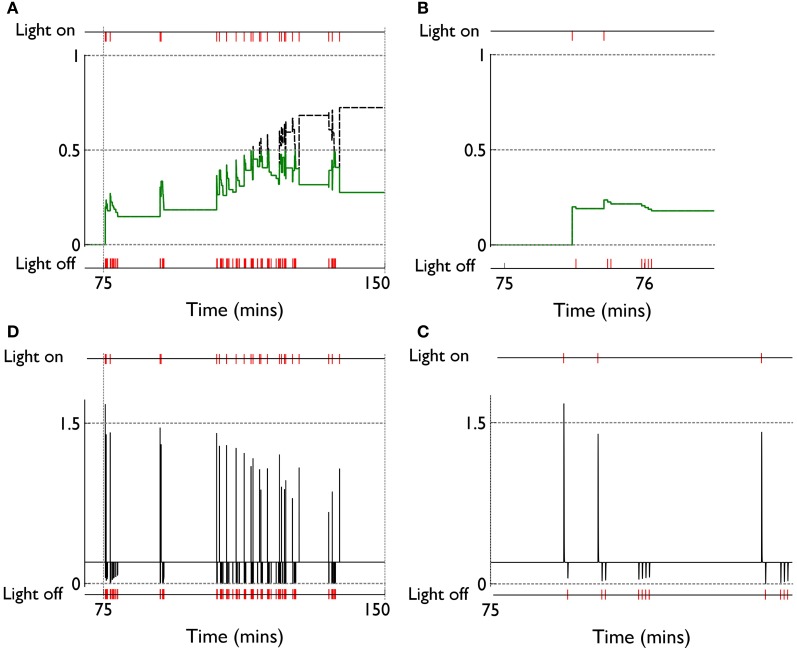
**Signals governing learning in pDA models with novelty salience and prediction, undergoing variable interval training. (A)** Shows the novelty salience (solid green line) and prediction signal (dashed black line) during the response contingent (RC) phase in a similar way to that used in Figure 5, but here the symbols have been omitted. **(B)** Is similar to **(A)**, but for a smaller temporal window immediately after the onset of the RC phase. **(D)** Shows the phasic dopamine signal corresponding to the events in **(A)**. **(C)** Is similar to **(D)** and relates to events in panel **(B)**.

We can see, mechanistically, the reason for the weight changes by examining the dynamics of the reinforcement signal (light flash), the prediction model, and resulting dopamine signal. These signals are shown in Figure 11. It is apparent that there are many more dopamine “dips” (negative prediction errors) than “bursts” (positive prediction errors) and so the factors *C*_BCM_ in the learning rule (Equation 10) are dominated by their low dopamine values. For D1/D2-MSNs this is positive/negative, respectively (Figure 7), which is also reflected in θ_BCM_. In addition, the high novelty salience in cortex causes high activity 〈*y*^2^〉 in the MSNs, thereby amplifying θ_BCM_ and any consequent effects on learning. These signs and magnitudes of θ_BCM_ lead to LTD/LTP for D1/D2-MSNs being likely (as θ_BCM_ appears in the factor (*y* − θ_BCM_) in the learning rule). This pattern of learning has computational and ethological consequences taken up in the section 4.

### 3.3. Phasic dopamine promotes plasticity in fixed-ratio training consistent with action learning in striatum

**Figure 12:**
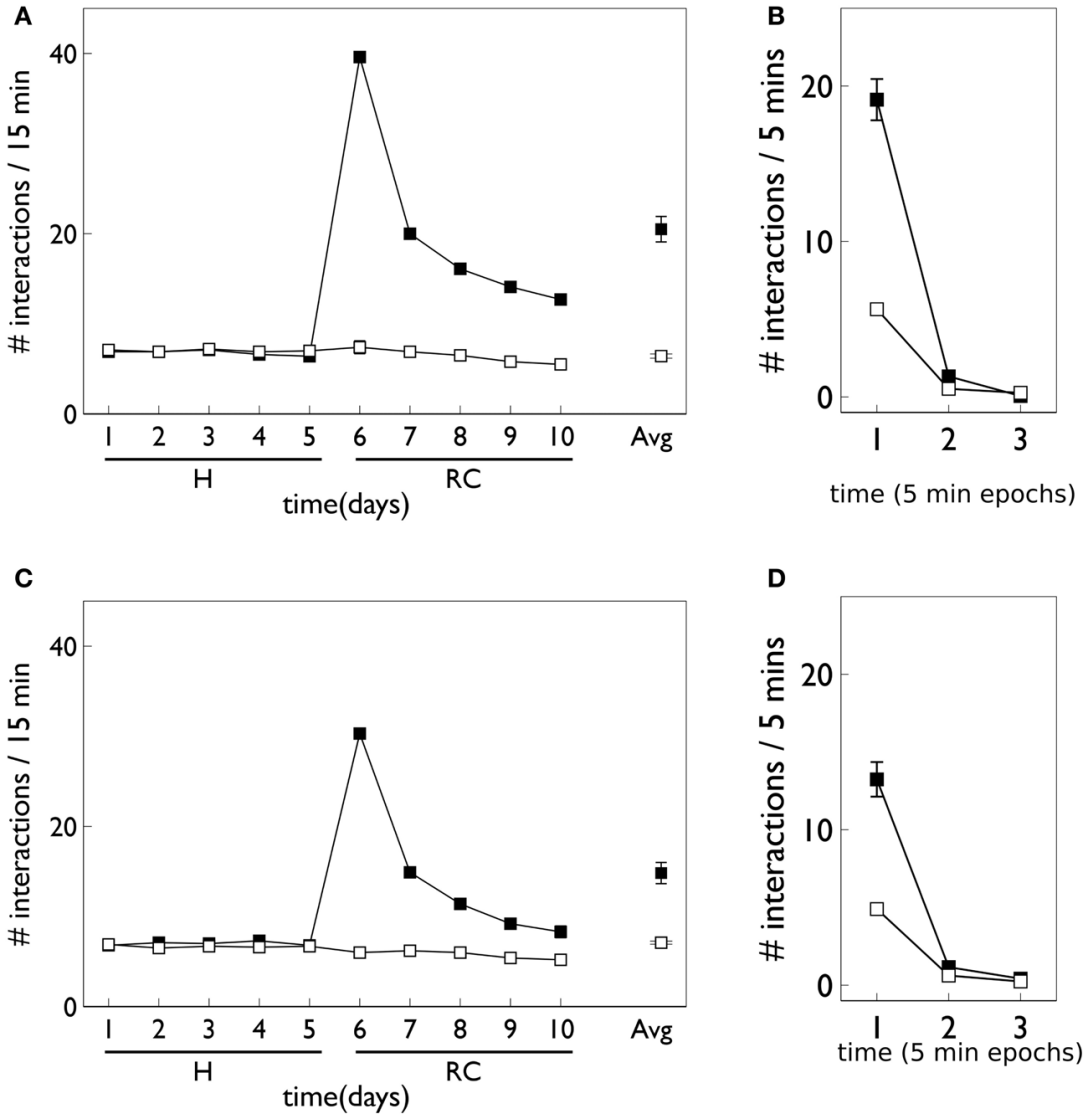
**Behavior of an agent with novelty salience and feature prediction performing the block-bumping experiment with fixed-ratio training.** All panels have the same significance as their counterparts for variable interval training in Figure 9. Thus, **(A,B)** are for pDA models, whereas **(C,D)** are for no-pDA models.

Figure 12 shows the behavioral responses of the robot in the fixed-ratio (FR) experiments. The results are qualitatively similar to those for VI training but there are fewer active responses and, unlike the VI behavior, the peak response occurs on the first day of the response contingent phase. This prediction was borne out by the study of Lloyd et al. (2012)—see Figure 3C. Within a session, the number of active responses declines more steeply than the corresponding VI data. This is similar to the *in vivo* data (Figure 3C) although the latter does not show such a tight clustering in the first epoch, with some residual responding at the end of the session.

The pDA and no-pDA models have similar behavior but the former shows somewhat more active responses (especially on the first response contingent day). This is quantified in the (rounded) ratios *r*_peak_ which are 6 and 4, respectively. These are both smaller than the values for the VI experiment, and have a different rank order (that for pDA is larger for FR, but is smaller for VI).

**Figure 13:**
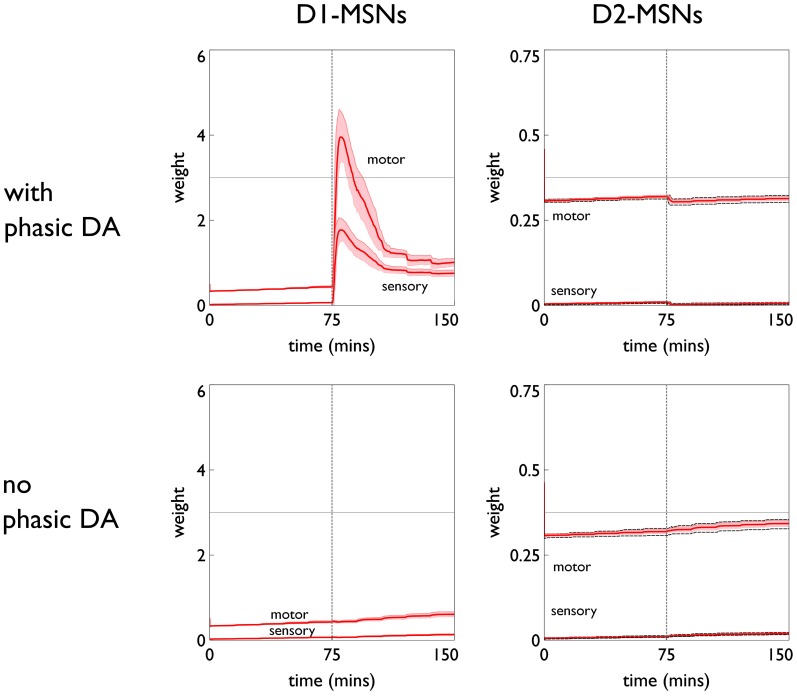
Weight trajectories w^*^(*t*) for the active response channel, in models with novelty salience and prediction, undergoing fixed-ratio (FR-1) training. All panels have the same significance as their counterparts for variable interval training in Figure 10. Note the different scale for D1 and D2-MSNs.

The similarity in behavioral response over the pDA, no-pDA variants is in stark contrast to the difference in weight trajectory (Figure 13).

The pDA model shows a very large transient change in the D1-MSN weights (both motor and sensory) with a substantial final change compared to initial baseline. This plasticity is clearly responsible for the extra activity in the response contingent phase compared to that for no-pDA models. None of the other weight trajectories show significant variation.

**Figure 14:**
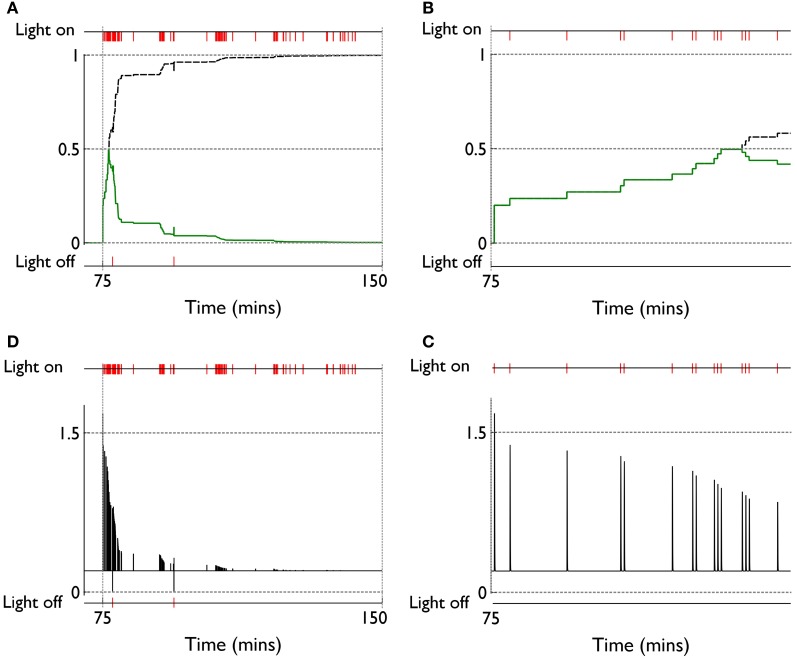
**Internal variables and phasic dopamine signals for a model with novelty salience and prediction undergoing fixed interval training. (A,B)** Show novelty salience and the prediction signal, and are counterparts to Figures 11B,C. **(C,D)** Show phasic dopamine and are counterparts to Figures 11A,D.

The clustering of active response in day 6 and the transient weight change associated with this are explained by reference to the prediction, novelty salience and dopamine signals shown in Figure 14. Thus, there is a large increase in novelty salience in the first part of the response contingent phase (panel **A**) but this is short lived as the prediction becomes reliable. This is made possible, of course, by the reliable delivery of the reinforcement. The phasic dopamine reflects this, and is almost always signalling positive reinforcement errors (the very few occasions for which this is not the case, are caused by failure of the robot to bump properly against the block). High levels of (phasic) dopamine occurring during these events is associated with negative values of *C*_BCM_ for D1-MSNs in the learning rule [Equation (10), and Figure 7]. This implies θ_BCM_ < 0 too, so that there is a likelihood of LTP as observed.

## 4. Discussion

### 4.1. Main results and their interpretation

We have used the embodiment of a biologically plausible model of intrinsically motivated operant learning (action discovery) to explore the possible roles of cortical salience, cortico-striatal plasticity in basal ganglia, and phasic dopamine therein. The embodiment allowed us to use behavioral data (Gancarz et al., 2011) to constrain the model, and our core model component was sufficiently biologically plausible to take advantage of a new framework for dopamine-dependent cortico-striatal plasticity constrained by a comprehensive suite of physiological data (Shen et al., 2008; Gurney et al., 2009).

In seeking an understanding of action discovery, we are primarily interested in the ethological situation in which the required action reliably produces the desired outcome; in the current context this is what has been referred to as the FR1 schedule. However, the data we have access to (Gancarz et al., 2011) concern a VI schedule. We have shown that cortico-striatal plasticity alone is insufficient to account for the increased active response in this data. In order to successfully model the behavioral data, we were therefore forced to consider the other possible contribution to more prolific action selection—an increase in the salience of the action request. Thus, we proposed that the sensory contribution to the action request for block interaction is enhanced by inheriting the novelty of any surprising phasic outcome associated with the target block. To incorporate this “novelty salience” we deployed a simple phenomenological model of prediction of the phasic outcome and its influence on the salience. We also used the prediction model to describe the dynamics of the sensory prediction error signal manifest in phasic dopamine.

With these components in place, the main trends in the behavioral data of the *in vivo* experiment could be replicated. Moreover, there was a somewhat counterintuitive result that there were fewer active responses with phasic dopamine than without. Further, the relative number of responses (active/inactive) in the data was better approximated by the inclusion of phasic dopamine. This difference could be explained by noting the preponderance of phasic dopamine dips in the VI schedule, the consequent weight dynamics, and their interpretation in the context of selection (GO) and NO–GO pathways in basal ganglia.

The attenuation of activity by dopamine mediated plasticity in the VI schedule is ethologically rational. The outcome in VI training is highly unpredictable and it is therefore fruitless for an intrinsically motivated agent to waste resources in attempting to build a model of agency. This notion has been formalized by Schmidhuber (2009) who argues that agents seek to compress information about their world (equivalent to our internal model building) and failure to see progress in this regard will cause them to disengage with the situation. Attempts to persist in doing so could lead to irrelevant and “superstitious” behavior (Pear, 1985). The dopamine mediate plasticity appears to prevent just this scenario. In addition, the failure of the D1-MSNs to show strong LTP would mitigate against the possibility that these neurons could learn to encode a match between their synapses and cortical representations of the new action request.

We carried over the notion of novelty salience to the FR1 schedule; there is no reason to suppose that the mechanisms for prediction and novelty salience generation suddenly become muted because the statistics of the stimulus are changed. The result was a strong increase in active responses on the first day of the response contingent phase. Overall activity during this time was, however, less than that for the VI schedule. Both these predicted features were shown in a recent *in vivo* study (Lloyd et al., 2012).

In contrast with the simulated VI result, phasic dopamine in FR learning enhanced the activity level with respect to the no-dopamine control. Further, much of the interaction occurred early in the session (also broadly in line with the *in vivo* data) and subsequent epochs within a session showed little interaction with the blocks. Activity is refreshed somewhat at the start of each day, which can be attributed to the dishabituation of block salience between days.

The rapid increase in, and subsequent decline of, responding with the novel situation is exactly what we would require with our repetition bias hypothesis. The results suggest that, while the behavioral repetition is due to a combination of novelty salience *and* plasticity (there is more responding with phasic dopamine) the bulk of this effect is caused by the novelty salience. We therefore predict that lesioning systems that may be responsible for developing novelty salience should severely compromise action-outcome learning (see discussion of novelty below).

We also predict a residual, persistent elevation of the number of active responses at the end of the response contingent phase, compared to that at the end of the habituation phase. There is some indication of this in the study of Lloyd et al. (2012) but further experiments would help confirm or falsify this outcome. In the event that it is true, this may be interpreted as the “bumping-into-the-red-block” action having acquired the status of a preferred action or *affordance* (Gibson, 1986; McGrenere and Ho, 2000). Thus, we suppose, along with Cisek (2007), that affordances become what we have dubbed “action requests,” subject to competitive selection by basal ganglia.

The weights in FR learning show strong LTP in D1-MSNs consistent with the encoding of the action in basal ganglia via synaptic-afferent matching. There is a marked peak during the early sessions of the response contingent phase (promoting repetition bias) before a decline to an equilibrium level which is elevated with respect to the initial value. It is only in the FR schedule with phasic dopamine that we see such a substantial weight increase and so we deem these conditions to be necessary for action learning.

### 4.2. Relation to other work

There have been many attempts in disembodied models to describe the role of phasic dopamine in animal learning. Most of these use some kind of RL technique and, typically something like the temporal difference (TD) algorithm (Sutton and Barto, 1998) or variants therein—for a recent review see Samson et al. (2010). These machine learning algorithms require an explicit representation of *value* as the expected sum of rewards over some predefined trial or epoch. However, no such representation prevails in our model. Further, in the TD-like schemes, there is usually a fine-grained representation of time supporting a correspondingly rich state-based description of the environment; we have no recourse to such a description. Like TD, our model uses a prediction error. However, this error has a quite different form from that in TD, is used in a quite different way to update the weights, and the update rule for the prediction is different.

Another hallmark of the general RL models is their emphasis on obtaining optimal behavior driven by explicit biological reward. In contrast we have emphasized the concept of novelty and sensory prediction as a primary source of reinforcement in the learning rule. Novelty has been used in TD-learning models of learning under phasic dopamine, appearing in the guise of “novelty bonuses.” Kakade and Dayan (2002) show how such a model may be used to enhance the explanatory power of the basic TD-learning approach, but the very term “bonus,” is used advisedly here to imply that novelty is an “add on,” and that optimality of reward acquisition is the primary feature of the algorithms. We revisit the issue of whether dopamine encodes reward or sensory prediction errors in section 4.3 where we give a possible resolution of this apparent dichotomy. The model of Kakade and Dayan (2002) is also unable to supply an explanation (even at an algorithmic level) of the intrinsically motivated learning seen in the study of Gancarz et al. (2011) because it does not address the issues of novelty salience that we have found necessary in our model.

In more biologically plausible (but still disembodied) approaches, many models of RL in basal ganglia use the actor-critic framework (Barto, 1995; Suri and Schultz, 1998, 1999). However, the applicability of this framework to the study of learning in basal ganglia has been questioned on the basis of its biological plausibility (Joel et al., 2002). In contrast, our approach does not rely on the actor-critic scheme. Further, many of the RL models that attempt to explain dopamine dynamics and learning in basal ganglia use the TD algorithm (Suri, 2002) which was noted above to be quite different from our approach. In a recent review, Frank (2011) notes several biologically plausible models of dopamine modulated learning in basal ganglia (Brown et al., 2004; Frank, 2005, 2006). However, these models do not address the problems surrounding intrinsically motivated learning and will therefore not seek to understand the automatically shaped, phasic period of repetition bias under the control of surprise or novelty, signalled by phasic dopamine. One recent model (Hazy et al., 2010) does note the possible utility of encoding “novelty value” in the phasic dopamine signal as well as reward, but this model is at a somewhat abstract level without explicit reference to basal ganglia components.

There are very few *robotic* models of operant learning that seek to explain the role of phasic dopamine. The model by Baldassarre et al. (2013) explores several of the issues in our general framework but at higher level of abstraction. It has a less physiologically constrained learning rule, several *ad hoc* mechanisms in place to test general computational hypotheses (such as repetition bias), the basal ganglia component is less well detailed, no mention is made of novelty salience, and there is no behavioral data against which it is constrained. Nevertheless, this model does integrate many of the features in the general scheme outlined in the Introduction (Figure 1A) and show how they may be deployed in concert with each other to achieve intrinsically motivated learning of actions.

The model of Sporns and Alexander (2002) (see also Alexander and Sporns, 2002) uses properties ascribed to the animal dopaminergic system in its learning, but the model architecture is rather abstract and has no reference to basal ganglia and cortico-striatal connectivity. In contrast to our own, this model also emphasizes the precise temporal representation of reward prediction reminiscent of the TD learning algorithm. An explicit use of TD learning was invoked by Pérez-Uribe (2001) but again, this model used a somewhat abstract actor-critic architecture. The model by Thompson et al. (2010) emphasizes limbic loops through the basal ganglia which deal with genuine reward-related behavior rather than intrinsically motivated behavior (hence no mention of novelty salience) and, again, it uses a different approach to learning. Khamassi et al. (2011) have recently described a robot model of learning with dopamine signalling prediction errors based on salient phasic events but their emphasis is on plasticity in cortico-cortical rather than cortico-striatal connections, with the aim of storing action values in anterior cingulate cortex (ACC).

### 4.3. Novelty, dopamine, and reward

One of the key ideas in our general framework is that intrinsically motivated action discovery is tightly bound up with the notion of novelty; new and unexpected objects or situations cause an agent to investigate them and discover operant contingencies. We have invoked two kinds of novelty in the present model: stimulus (object) novelty and surprise (phasic outcome). We have identified the detection of the latter with the SC and have noted the intimate link between the detection of surprise and release of phasic dopamine (Comoli et al., 2003; Dommett et al., 2005). However, the detection of novelty salience remains unresolved. Several brain areas have implicated in the detection of novelty and are candidates for this process including: lateral prefrontal cortex, anterior insular and anterior temporal cortex, parahippocampal cortices, and the hippocampal formation itself (Ranganath and Rainer, 2003). In regards to the latter, Kumaran and Maguire (2007) have proposed that the hippocampus acts as a comparator between prediction and perception, while Lisman and Grace (2005) have noted the link between hippocampus and midbrain dopamine systems in novelty detection. Using fMRI studies in humans, Bunzeck and Düzel (2006) have also demonstrated how stimulus novelty can drive the activation of dopamine neurons. However, when elicited by object novelty (rather than the surprise of an outcome) phasic dopamine may be more potent in facilitating learning in the structures which may encode the prediction models—namely areas like the hippocampal complex and prefrontal cortex (Lisman and Grace, 2005; Bunzeck and Düzel, 2006)—rather than motor and associative territories of striatum.

The preceding discussion has highlighted the ubiquity of phasic dopamine as an encoder of novelty and, consistent with this, is a recurrent theme in our work that dopamine is a *sensory* prediction error. However, there is a substantial literature arguing for its role in encoding reward (for recent review see Schultz, 2010). Thus, several studies (Fiorillo et al., 2003; Tobler et al., 2005; Morris et al., 2006; Roesch et al., 2007) have shown that, with well trained animals, size of reward or its probability of delivery reward associated with unpredictable phasic cues produced phasic dopamine responses which reflected the expected amount of reward. This is often cited as strong evidence that phasic dopamine is signalling *reward*-prediction error. However, one possible resolution of this apparent conflict is to suppose that dopamine encodes a sensory prediction error which may be *modulated* by reward value. This can occur because repeated delivery of reward is known to sensitise primary sensory areas including: visual cortex (Weil et al., 2010), somatosensory cortex (Pleger et al., 2008), and SC (Ikeda and Hikosaka, 2003). Thus, using an abbreviated form of our prior notation, let *y*_*f*_ and y^*^_f_ be representations of a sensory feature and its prediction, respectively, and let *S*_R_ be a *reward sensitization* of *y*_*f*_ under extensive training (as typically deployed experimentally). We now hypothesise (Gurney et al., 2013) that phasic dopamine encodes

(13) $$e=S_{\text{R}}\left(y_{f}-y_{f}^{*}\right)$$

Notice that *e* can still be thought of as a *sensory* prediction error—there is no mention of a difference between *observed* or its prediction, as such. The stimulus feature has been “tagged” with additional value but the difference is fundamentally one between sensory features and their prediction. This idea can accomodate a recent theory by Bromberg-Martin et al. (2010) in which two classes of dopamine neuron are identified. In one class, dopamine neurons encode *motivational value*—the conventional idea that dopamine signals prediction errors of rewarding/aversive stimuli with positive/negative-going responses, respectively. A second class of neuron encode *motivational salience* with positive responses irrespective of the rewarding/aversive significance of the predicted stimulus. However, both classes of dopamine neuron signal “alerting” or unpredicted sensory cues. This classification is consistent with Equation (13) if we allow two cases in which *S*_R_ is either a signed quantity, encoding rewarding/aversive value, or simply the absolute magnitude of this quantity.

### 4.4. Future directions

The action discovery used in our model is of the simplest kind; a given “atomic” movement (bump a block) has been paired with a context (the red block in this arena) to facilitate the prediction of the outcome (light flash above the block). However, in general we can imagine more complex combinations of action components may need to be assembled with the context. For example, the agent may not know how to perform a bumping sequence (move forward, then back and slow down), in which case it has to explore possible combinations of atomic movements at a lower level of granularity and chunk them together to make the new action. These lower level action components may also have to occur simultaneously rather than sequentially (e.g., bumping may require extending an effector as well as moving forward). Modeling the discovery of these more complex action assemblies is an important next step.

One of the requirements of a multi-component action model would be a true distributed representation of motoric commands. Even with a single atomic movement this is most likely encoded in a more plausible way a vector of command components. Further work would test the learning rule with these higher dimensional vector inputs. This was the approach taken in our spiking model of plasticity (Gurney et al., 2009) and, indeed, one possible progression of the model would be to embed the spiking model of MSNs into the larger basal ganglia model used here. This multi-scale model would enable a closer examination of the finer details of the learning rule as originally conceived. Finally, we aim to test experimentally, predictions about the expected behavior of animals in an FR learning schedule with dopamine lesions.

### Conflict of interest statement

The authors declare that the research was conducted in the absence of any commercial or financial relationships that could be construed as a potential conflict of interest.

## Appendix

### Details of biomimetic core model

We give details here of the equations defining the biomimetic core model which were not given in the main text. In most cases this amounts to identifying the form of the net input *I* in Equation (5), and parameterizing the output function (Equation 6). In what follows, indices refer to action channels.

#### Basal ganglia

Sensory, and motor cortical output are denoted by *y*^*S*^_*i*_, *y*^*M*^_*i*_, respectively. The tonic dopamine level λ = 0.2.

$$\begin{array}{l} \text{Striatum D1: }I_{i}^{\text{D1}}=(w_{i}^{\text{S},\text{ D1}}y_{i}^{\text{S}}+w_{i}^{\text{M},\text{ D1}}y_{i}^{\text{M}})(1+\lambda )\\ \text{ with initial weight values; }w_{i}^{\text{S},\text{ D1}}=0,\\ \;w_{i}^{\text{M},\text{ D1}}=0.45\\ \;y_{i}^{\text{D1}}=L(I_{i}^{\text{D1}},\;0.1)\\ \text{Striatum D2: }I_{i}^{\text{D2}}=(w_{i}^{\text{S},\text{ D2}}y_{i}^{\text{S}}+w_{i}^{\text{M},\text{ D2}}y_{i}^{\text{M}})(1-\lambda )\\ \text{ with initial weight values; }w_{i}^{\text{S},\text{ D2}}=0,\\ \;w_{i}^{\text{M},\text{ D2}}=0.45\\ \;y_{i}^{\text{D2}}=L(I_{i}^{\text{D2}},\;0.1)\\ \text{ STN: }I_{i}^{\text{STN}}=0.4(y_{i}^{\text{S}}+y_{i}^{\text{M}})-0.2y_{i}^{\text{GPE}}\\ \;y_{i}^{\text{STN}}=L(I_{i}^{\text{STN}},\;-0.25)\\ \text{ GPe: }I_{i}^{GPe}=0.3\sum_{i=1}^{3}y_{i}^{\text{STN}}-0.9y_{i}^{\text{D2}}\\ \;y_{i}^{\text{GPe}}=L(I_{i}^{\text{GPe}},\;-0.2)\\ \text{ GPi/SNr: }I_{i}^{\text{GPi}}=0.3\sum_{i=1}^{3}y_{i}^{\text{STN}}-0.7y_{i}^{\text{D1}}-0.4y_{i}^{\text{GPe}}\\ \;y_{i}^{\text{GPi}}=L(I_{i}^{\text{GPi}},\;-0.12) \end{array}$$

#### Thalamus and brainstem

$$\begin{array}{l} \text{ TRN}:\;I_{i}^{\text{TRN}}=y_{i}^{\text{M}}+y_{i}^{\text{VL}}\\ \;y_{i}^{\text{TRN}}=L(I_{i}^{\text{TRN}},\;0)\\ \text{VL Thalamus}:\;I_{i}^{\text{VL}}=0.9y_{i}^{\text{M}}-y_{i}^{\text{GPi}}\\ \;-0.01y_{i}^{\text{TRN}}\left(1-0.11\sum_{j\neq i}y_{j}^{\text{TRN}}\right)\\ \;y^{\text{VL}}=L(I_{i}^{\text{VL}},\;0)\\ \text{ Brainstem: }I_{i}^{\text{BS}}=y_{i}^{\text{M}}(1-1.5y_{i}^{\text{GPi}})\\ \;y_{i}^{\text{BS}}=L(I_{i}^{\text{BS}},\;0) \end{array}$$

The action is behaviorally enacted if *y*^BS^_*i*_ > ϕ (recall ϕ = 0.5).

#### Cortex

For the sensory cortex, the input *c*_*i*_ is provided by the salience generation process (section 2.5.2)

$$\begin{array}{l} I_{i}^{\text{S}}=c_{i}\\ y_{i}^{\text{S}}=L(I_{i}^{\text{S}},\;0) \end{array}$$

For motor cortex, we consider two classes of action representation. For the “explore” action, arbitrarily assigned as channel 1

$$\begin{array}{l} \;I_{1}^{\text{M}}=0.75y_{1}^{\text{S}}+0.89y_{1}^{\text{VL}}\\ y_{i}^{\text{M}}=L(I_{i}^{\text{M}},\;0) \end{array}$$

For the block-interaction channels (*i* = 2, 3), we incorporated a recurrent, self reinforcing connection if the action is currently selected.

$$\begin{array}{l} I_{i}^{\text{M}}=0.75y_{i}^{\text{S}}+0.89y_{1}^{\text{VL}}+0.005y_{i}^{\text{M}}H(y_{i}^{\text{BS}}-\phi )\\ y_{i}^{\text{M}}=L(I_{i}^{\text{M}},\;0) \end{array}$$

where *H*() is the Heaviside step function and ϕ is the same threshold used in selecting behavior in brainstem (see “Thalamus and Brainstem,” above). The self-recurrence here plays a similar role to the “busy signal” used by Prescott et al. (2006) to ensure correct execution of fixed action patters (FAPs) which should not time-out before their completion. This signal was driven explicitly by an internal clock and knowledge of the FAP duration. In contrast, we have taken a slightly different approach, which is more neurally plausible and does allow for interruption of the action by a very highly salient competitor. In this way we have something more akin to a soft-action pattern (SAP) process.
